# Infection with Epstein Barr virus increases risk of multiple sclerosis

**DOI:** 10.1038/s43856-022-00076-w

**Published:** 2022-02-07

**Authors:** Katharine Barnes

**Affiliations:** Communications Medicine, https://www.nature.com/commsmed/

## Abstract

The causes of multiple sclerosis are unclear, but viral infection has been proposed as a possible trigger. A longitudinal analysis in a large cohort published in *Science* found that the risk of multiple sclerosis increased 32-fold following infection with Epstein-Barr virus (EBV).

**Figure:**
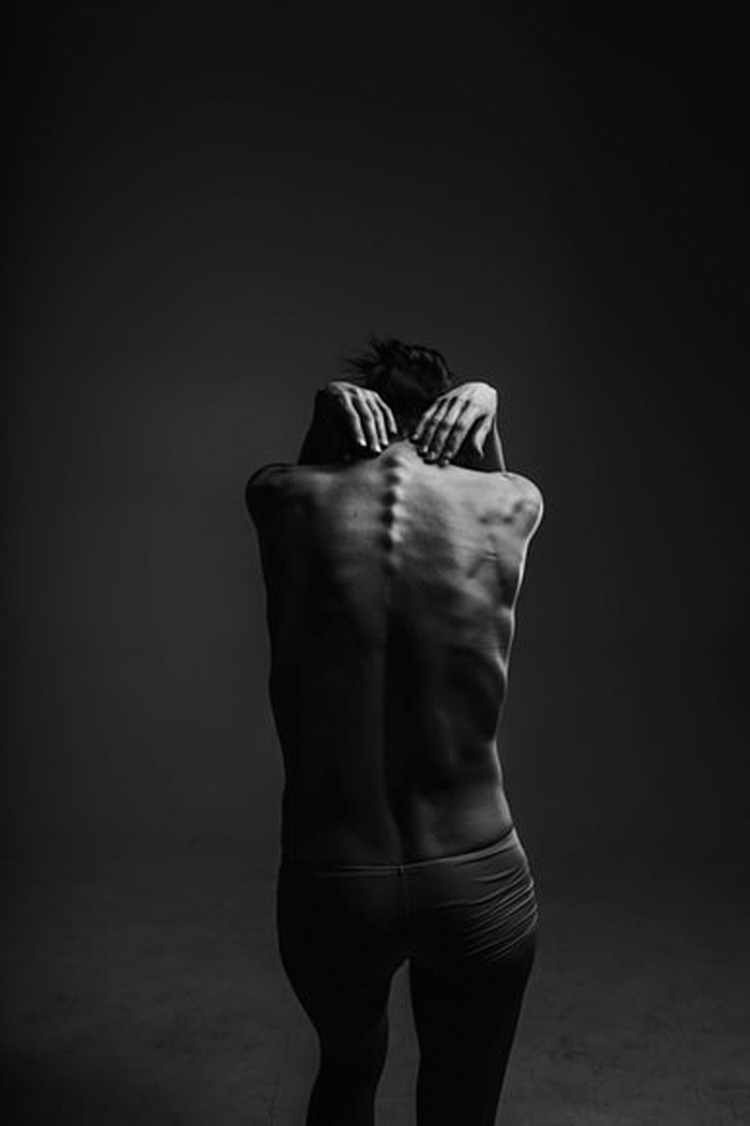
Unsplash

Multiple sclerosis (MS) is a debilitating chronic autoimmune disease in which there is demyelination of the neurons within the brain and spinal cord. The immune system is involved in the demyelination process, and it has previously been proposed that demyelination is initiated by viral infection. Human EBV remains present in the body after infection and has been found in some MS demyelinated lesions. It has thus been proposed that infection with EBV could initiate MS.

Bjornevik et al. identified cases of MS in a cohort of over 10 million active-duty US military personnel between 1993 and 2013^1^. 801 cases of MS were matched to randomly selected individuals without MS but with similar demographic and service details. Blood serum samples that had been taken at regular intervals were screened for EBV.

All but one of the 801 cases of MS occurred in individuals who had been infected with EBV. On average, individuals who developed MS became symptomatic 5 years after infection with EBV. 43% of individuals who did not develop MS were EBV-negative.

Antibodies against cytomegalovirus (CMV) were also detected to determine whether other infections were associated with development of MS. In contrast to results with EBV, MS risk was lower amongst CMV-positive individuals compared to CMV-negative individuals. Participants were also screened more generally for viral infections, and evidence of viral infection was found to be similar for cases and controls both before and after EBV infection. This suggests that the development of MS is not associated with a more general susceptibility to viral infections.

The authors further investigated the relationship between EBV infection and MS by measuring concentrations of neurofilament light chain in the blood serum samples. Neurofilament light chain levels are known to increase up to 6 years before clinical MS onset and so have been proposed to be a more accurate indicator of the initiation of the disease process. Neurofilament light chain levels were low in EBV-negative individuals, but increased following EBV infection.

While other factors have been identified that increase the risk of developing MS, the biggest increase in risk that has been identified previously was just 3-fold. In contrast, the extremely low incidence of MS seen in EBV-negative individuals in this study suggests that most MS cases are caused by EBV. This also provides an explanation for the effectiveness of anti-CD20 monoclonal antibodies as a treatment for MS, as these antibodies reduce circulating memory B cells, which retain EBV after initial infection. Because a link has been found between EBV and MS, other interventions that prevent EBV infection or treat EBV could also reduce incidence of MS. For example, preventing EBV infection by vaccination or treating with other anti-virals that target EBV.
